# Clinical Implications of Kratom (*Mitragyna speciosa*) Use: a Literature Review

**DOI:** 10.1007/s40429-023-00478-3

**Published:** 2023-05-12

**Authors:** Elisabeth Prevete, Kim Paula Colette Kuypers, Eef Lien Theunissen, Gianluca Esposito, Johannes Gerardus Ramaekers, Massimo Pasquini, Ornella Corazza

**Affiliations:** 1 Department of Human Neurosciences, Sapienza University of Rome, Viale Dell’Università 30, 00185 Rome, Italy; 2 Department of Neuropsychology and Psychopharmacology, Faculty of Psychology and Neuroscience, Maastricht University, P.O. Box 616, 6200 MD Maastricht, the Netherlands; 3 Department of Psychology and Cognitive Science, University of Trento, Corso Bettini, 84, 38068 Rovereto, Italy; 4 Department of Clinical, Pharmacological and Biological Sciences, University of Hertfordshire, College Lane, Hatfield, AL10 9AB UK

**Keywords:** Kratom, Mitragynine, *Mitragyna speciosa*, Clinical implications, Therapeutic use, Adverse effects

## Abstract

**Purpose of Review:**

This work aims to provide an up-to-date review of the preclinical and clinical scientific literature on the therapeutic value of kratom to better understand the underlying mechanisms related to its use and inform future therapeutic applications.

**Recent Findings:**

A growing number of studies, mainly of cross-sectional nature, describe the widespread use of kratom by individuals to self-treat pain, psychiatric symptoms, and substance use disorders (SUD) outside a controlled clinical setting. Preclinical evidence suggests kratom is effective as an analgesic agent and might decrease the self-administration of other drugs. A randomized controlled trial has further supported kratom’s therapeutic value as an analgesic. Investigations in nonclinical samples of long-term kratom users also indicate its therapeutic benefit in managing SUD symptoms (e.g., craving) and long-term or acute symptoms (e.g., withdrawal) for alcohol, opioids, and other illicit drugs. However, episodes of kratom-related intoxications have also been reported, often due to the adulteration and the contamination of kratom products mainly sold online or mixed toxicities when consumed outside clinical and traditional settings.

**Summary:**

Evidence on the clinical implications of kratom use is still limited and uncertain, with kratom research constantly evolving. Therefore, further randomized trials are needed.

## Introduction 

### Clinical Background

Over the past decade, kratom (*Mitragyna speciosa* Korth.) has widely spread from its Eastern native regions (Malaysia, Thailand, and Indonesia) to the West, especially in Europe and the United States (US) [1, 2••]. It is mainly used by white adults (ages 30–50 on average) who are well-educated and employed at least part-time (e.g., [3, 4••]). Estimates of kratom users largely differ, but millions of people have been using this plant in the US where it is federally legal in all the constituent states apart from Alabama, Arkansas, Indiana, Vermont, Wisconsin, and Rhode Island [1, 2••]. Kratom is sold in various formulations (i.e., capsules, resin, tinctures, powder) in headshops and on the internet [1, 2••]. Recent evidence has shown that kratom products are becoming quite popular also in darknet markets [5]. Kratom is used recreationally as a plant-based substance by some users. Its effects are described as psychostimulant in small dosages of up to 5 g of plant material and similar to opioids at higher doses of approximately 5 to 15 g [1, 6]. However, its native use in South Asia has been associated with the nonmedical self-treatment of several conditions (i.e., substance use disorder (SUD) symptoms, such as opioid and other drugs’ withdrawal, pain, and improving energy) described even in the West, as well as hypertension, stomach ailments, diarrhea, infections, and diabetes, among others [1,7,8].

### Preclinical Data

From a pharmacological perspective, mitragynine (MG) is the main lipophilic alkaloid present in kratom. Its psychoactive metabolite 7-hydroxy-mitragynine (7-HMG) is widely studied in preclinical models for its analgesic properties, while few studies have been conducted on the other alkaloids present in the plant. Another alkaloid, speciociliatine, has also been recently studied [9••]. MG possesses a poly-pharmacological profile and a primary opiate receptor activity, with preliminary evidence suggesting the possibility of mu-opioid receptor (MOR) activity without β-arrestin-2 recruitment in the signaling pathway [10•]. Recent evidence has shown that high doses (between 20 and 400 mg/kg) of oral MG do not exert respiratory depression compared to oxycodone in animal models [11]. However, information related to the safety profile of MG is still quite limited. Furthermore, in February 2018, the Food and Drug Administration (FDA) expressed concerns about the abuse potential of MG and 7-HMG contained in unregulated kratom-related products, highlighting that no medical indication has been approved for such herbal supplements [12]. Similar concerns have emerged in Europe, where kratom remains illegal in various countries (e.g., Sweden, Poland, Romania, and Denmark, among others) [13].

With the increasing scientific interest in the balance between kratom’s risks and potential therapeutic use, the present paper aims to provide an up-to-date review of the preclinical and clinical scientific literature on this topic to understand the underlying mechanism of kratom use and inform future therapeutic applications. The key objectives of our search were to (a) evaluate evidence suggesting kratom’s therapeutic value from a clinical perspective, including both anecdotal information and (pre)clinical evidence; (b) identify the clinical issues/health hazards linked to kratom use; and (c) understand if any information is available on the clinical consequences of kratom use in naïve users.

## Methods

An exploratory literature search was performed between May 2022 and January 2023 in PubMed, Medline, Google Scholar, ScienceDirect, and Cochrane. “*Kratom*”, “*mitragynine*”, and “*Mitragyna speciosa*” were used as keywords to carry out the databases’ searches. We excluded all the duplicates and commentaries. Studies considered recent as published within the past 5 years were considered for analysis. Particular attention was given to all the (pre)clinical studies also if published outside this timeframe as limited clinical evidence is available.

## Narrative Review Results

### Evidence Suggesting Kratom’s Therapeutic Value from a Clinical Perspective

#### Anecdotal Information

There is an ongoing debate in the scientific community to understand the complex profile of kratom and its alkaloids regarding safety, health issues, and potential medical benefits. Anecdotal data, mainly from its native countries, suggest it has therapeutic value as an aid in several domains. Data from qualitative reports, e.g., social media analyses [14, 15] and large-scale surveys (e.g., [16••, 17••]), all document its widespread reported use in nonmedical settings for treating pain such as that related to the coronavirus disease 2019 (COVID-19) [18], cancer, multiple sclerosis, neuropathy and trigeminal neuralgia, fibromyalgia, headache, rheumatoid arthritis, and other inflammatory conditions (i.e., lupus, osteoarthritis, inflammatory bowel disease, complex regional pain syndrome, Ehlers-Danlos syndrome) [2••, 19]. Kratom use has also been reported as a substitute in nonmedical settings for dangerous drugs (i.e., methamphetamine, benzodiazepines, or heroin) or to mitigate the withdrawal from opioids, alcohol, and other illicit substances [20••, 21], especially among opioid poly-drug users [22]. Other underlying motivations for kratom intake include the improvement of sexual performance [23], coping strategies for negative emotional states [24], ameliorating psychiatric symptoms (i.e., anxiety, depression, insomnia, attention deficit hyperactivity disorder (ADHD), and post-traumatic stress disorder (PTSD) [e.g., 4••, 16••], and the enhancement of sociability, energy, and focus [14]. A recent case even reported its use in post-COVID insomnia [25].

#### Preclinical Evidence

Evidence emerging from the initial preclinical findings supports the anecdotal reports that kratom can be beneficial in treating pain and mental health–related conditions [26••]. In vitro and/or in vivo studies have shown that kratom and its alkaloids exert analgesic/antinociceptive effects as suggested by the increased latency of an antinociceptive response in the hot plate or tail-flick tests or in the model of inflammatory pain induced by acetic acid (27, 28••). Anti-allodynic efficacy in neuropathic pain has also been described [29••, 30•). Moreover, some preclinical models have suggested kratom’s antidepressant, anxiolytic, stress-mitigating [31••, 32•, 33] and antipsychotic effects [34•]. It has also emerged that kratom and its alkaloids exert gastroprotective, anti-inflammatory [35••, 36], antibacterial [37], antioxidant, antimutagen, and anticancer actions [38]. A recent preclinical study showed, for instance, that MG and speciociliatine acted as chemo-sensitizers for cisplatin and inhibited cell proliferation of nasopharyngeal carcinoma, malignant cancer [9••]. In another current study, MG has been shown to inhibit the enzyme acetylcholinesterase (AChE) involved in Alzheimer’s disease [39••]. It might also exert a lipolytic effect [40••] and antidiabetic action by inhibiting other biological enzymes (α-glucosidase, pancreatic lipase) when combined with the diabetes-2 drug (α-glucosidase inhibitor) acarbose [41••]. Preclinical evidence has also suggested its potential in treating (i) alcohol use disorder, alcohol withdrawal, and alcohol-seeking behavior, with a large therapeutic window (e.g., [42, 43••]); (ii) dependence and withdrawal from opioids (e.g., [28••, 44••]) without anxiogenic symptoms [45] and by improving simultaneously cognitive performance [46••]; and (iii) craving and addiction from methamphetamine [47] (for an overview of preclinical evidence and anecdotal data providing some evidence suggesting kratom’s therapeutic potential, see also Table 1).

**Table 1:** Summary of the limited evidence on kratom and its alkaloids’ potential medicinal effects as suggested in preclinical models and based on kratom benefits anecdotally claimed by users. *ND* not described

Action/effect	Preclinical evidence	Anecdotal evidence
Analgesic	√ [27, 28••, 29••, 30•]	√ [14, 16••, 17••, 19]
Antibacterial	√ [37]	ND
Antidepressant-like	√ [31••, 32•]	√ [4••, 16••, 17••]
Antidiabetic and lipolytic	√ [40••, 41••]	ND
Anti-inflammatory	√ [35••, 36]	√ [19]
Antioxidant, antimutagen, and anticancer	√ [9••, 38]	ND
Antipsychotic-like	√ [34•]	√ [4••, 16••, 17••]
Anxiolytic-like and stress mitigating	√ [31••, 33]	√ [4••, 16••, 17••]
Dependence on/withdrawal from alcohol	√ [42, 43••]	√ [17••, 20••]
Craving/addiction to methamphetamine	√ [47]	√ [17••, 21]
Dependence on/withdrawal from opioids	√ [28••, 44••, 45, 46••]	√[(17••, 20••, 22]
Gastroprotective	√ [36]	ND
Inhibition of acetylcholinesterase (Alzheimer’s disease)	√ [39••]	ND
COVID-19	ND	√ [18]
Coping strategies	ND	√ [24]
To enhance sociability, concentration, energy	ND	√ [14]
To improve sexual performance	ND	√ [23••]

In light of the anecdotal and preclinical findings, little data is available on kratom’s therapeutic potential.

#### Evidence from Clinical Studies

The discussion on the clinical implications of kratom use has been further enriched by the findings derived from some observational studies carried out in a traditional context (Malaysia or Thailand) among long-term/daily kratom users drinking daily several glasses of kratom juice/tea (between 2 and 6) [48, 49, 50, 51••]. In these cases, the estimated daily dose of MG ranges between 76 and 434 mg [52, 53], with no data on other alkaloids. Some of these studies have reported the association between long-term kratom use and health issues. This includes kratom’s impact on visual episodic memory and alteration of cholesterol level [54, 55], early stage of renal injury [50], dependence (which affects physical well-being if severe; [48, 56]), and craving with physical and psychological withdrawal symptoms [58, 59]. Such symptoms last about 3 days but are less severe than those described in the West or those related to classical opioids [57, 58, 59, 60]. However, until now, there is no evidence of kratom-related psychosis, social functioning alterations, long-term cognitive or biochemical/endocrinological impairment [53, 54, 61, 62, 63]. Furthermore, preliminary clinical data suggest kratom’s therapeutic potential as (i) an analgesic agent, with a randomized controlled trial (RCT) in kratom users showing enhanced pain tolerance after drinking kratom without any significant negative health consequences [51••], and (ii) a form of harm reduction, with kratom being reported to reduce regular drug (heroin, methamphetamine, amphetamine) use, opioids adverse effects, and HIV risk behaviors among illicit and opiates drug users [64, 65, 66]. Some evidence has also suggested that kratom possesses a favorable lipid profile and might be protective against metabolic syndrome, coronary heart, or cerebrovascular disease [63, 67, 68]. However, this data is in contrast with the slight increase in serum lipids linked to a higher frequency of kratom use reported by Leong Bin Abdullah et al. [69]. Finally, two studies evaluated the pharmacokinetic profile of MG in humans and suggest that it follows a two-compartment model with a 1-day terminal half-life [70••, 71]. As reported by the authors, such beneficial pharmacokinetic properties might be useful for its potential use as a medication (for a summary of clinical studies conducted on kratom users, see Table 2).

**Table 2:** Clinical studies conducted on kratom users in the traditional setting and kratom-related clinical consequences. Besides a limited number of older studies, the most recent clinical studies among regular kratom users in the last 5 years (2018–2022) have been reported

Reference	Objectives	(1) Study design (type of study) (2) Sampling (3) Sample size (*N* = , gender, mean age) (4) No drug-using control participants (*N* = , gender, mean age)	Main measures/outcomes	Kratom-related clinical implications	
				Health issues	Suggested therapeutic potential
[72]	To test kratom effects on muscular and mental fatigue	(1) Experimental design (interventional study) (2) ND (3) Volunteers (*N* = 5, M, ND) (4) ND	MG acetate (0.05 g; p.o.): Produced symptoms/giddiness, slight confusion, feeling of haziness, tenseness of muscles; choice reaction time/reduction; heat tolerance/less sensitivity to heat; ergographic record of the weight that the subject could lift/improvement in muscular work; steadiness test/increased steadiness; dotting test/slight increase in mental fatigue; electrical resistance of the skin/undetermined effect on blood vessels of the skin; range of vision/no significant effect; MS powdered leaves (0.65, 1.3 g p.o.): produced symptoms/similar to those produced by MG; choice reaction time/increase; heat tolerance/very slight change; ergographic record of the weight that the subject could lift/improvement in muscular work; dotting test/no significant effect; electrical resistance of the skin/dilatation of the blood vessels of the skin	Yes	Yes, less sensitivity to heat
[50]	To determine the urinary protein profile/renal effects of k-use	(1) Observational design (cross-sectional study) (2) Purposive and convenience sampling (3) Regular kratom users (*N* = 88, M, 44.5 years) (4) Healthy subjects (*N* = 83, M, 41 years)	Face-to-face interviews and semi-structured questionnaire; urine protein analysis, creatinine assay analysis, and toxicology test/higher concentration of urinary protein, PCR, and albumin in k-users	Yes, proteinuria (early stage of renal injury)	No
[67]	To examine the association between k-use and serum lipid level	(1) Observational design (cross-sectional study) (2) ND (3) Regular k-users (*N* = 285, M, 55.8 years) (4) Healthy subjects (*N* = 296, M, 55.7 years)	Structured questionnaire; blood exams and fasting lipid profile/association between k-use, high HDL level, and low TG, no significant difference for TC and LDL	No	Yes, to lower lipids
[68]	To evaluate the effect of traditional k-use on MetS	(1) Observational design (cross-sectional study) (2) Randomly sampling selection (3) Regular k-users (*N* = 285, M 78.6%, 55.77 years) (4) Healthy subjects (*N* = 296, M 29.7%, 55.73 years)	Blood exams/serum MG (14.7–380.7 ng/mL), FG, High HDL in k-users; BP/higher diastolic BP in k-users, no difference in systolic BP; MetS parameters/lower odds of MetS, smaller WC, lower TG and higher HDL in k-users	No	Yes, a potential protective effect against MetS
[48]	To evaluate QoL of k-users and its associated factors	(1) Observational design (cross-sectional clinical survey) (2) Snowball and purposive sampling (3) Regular k-users (*N* = 150, M, 34.4 years) (4) ND	WHOQOL-BREF/impairment in quality of life (only for severe dependence); KDS/mild to severe dependence	Yes	No
[61]	To investigate the prevalence of psychosis, its severity, and potential associations with k-use	(1) Observational design (cross-sectional clinical survey) (2) Snowball sampling (3) Regular k-users (*N* = 150, M, 34.42 years) (4) ND	MINI and BPRS/mild severe psychotic (positive) symptoms (6 users), not linked to variables (intake with diphenhydramine, duration, quantity, daily frequency) of k-use	No	No
[69]	To evaluate fasting lipid profile of k-users and its associations with k-use	(1) Observational design (analytical cross-sectional study) (2) Snowball sampling (3) Regular k-users (*N* = 100, M, 27 years) (4) Healthy subjects (*N* = 100, M, 29 years)	Blood exams: serum TG and HDL/no significant differences; serum TC and LDL/lower than healthy subjects, association between increased TC and age/higher average daily frequency of k-use; albumin, ALP, ALT, AST, TB, and DB/liver parameters within normal range	Yes, slight elevation of serum lipids for higher frequency of daily k-use	No
[56]	To investigate the impact of regular k-use on the QoL	(1) Observational design (cross-sectional study) (2) Snowball sampling (3) Regular k-users (*N* = 100, M, ND) (4) Healthy subjects (*N* = 100, M, ND)	WHOQOL-BREF/lower QoL physical health-, psychological-, and environment-related; KDS/presence of dependence (association between greater kratom dependence and lower psychological and environment QoL)	Yes	No
[73]	To investigate the prevalence of ECG abnormalities and QTc intervals in regular k-users	(1) Observational design (analytical cross-sectional study) (2) Snowball sampling (3) Regular k-users (*N* = 100, M, < 30 years—60 subjects—> 30 years—40 subjects) (4) Healthy subjects (*N* = 100, M, < 30 years—56 subjects—> 30 years—44 subjects)	Resting ECG/sinus tachycardia in k-users, no significant difference in other ECG abnormalities; borderline QTc interval (no association with k-use)	No	No
[49]	To assess the cardiovascular functioning and serum MG level of regular k-users	(1) Observational design (case series) (2) Snowball sampling (3) Regular k-users (*N* = 9, M, age between 18–43 years) (4) ND	Resting ECG/1 user—T wave inversion in inferior leads (III, aVF), prolonged QTc interval related to higher serum MG level; transthoracic echocardiogram/normal findings (except 1-left ventricular hypertrophy; 1- trivial tricuspid regurgitation with PASP of 10 + 5 mmHg); blood exams/mean serum MG level 10.3 mg/L, range 2.5–22.4 mg/L (higher in case of 4 or more glasses of kratom juice daily or prolonged QTc intervals)	Yes, dose-dependent prolonged QTc interval	No
[63]	To investigate biochemical and safety parameters of regular k-users	(1) Observational design (longitudinal study) (2) ND (3) Regular k-users (*N* = 5, M, ND) (4) ND	2 sessions (baseline and after 3 months)—blood exams and vital signs/no significant alterations; calcium, total protein, globulin levels/increase after 3 months; high TC and LDL at baseline and after 3 months; HDL/increase after 3 months; TG/decrease after 3 months	No	Yes, increased HDL and decreased TG
[74]	To investigate HRV indices of cardiac autonomic function and their association with k-use	(1) Observational (cross-sectional study) (2) ND (3) Regular k-users (*N* = 31, M, 51.32 years) (4) Healthy subjects (*N* = 19, M, 48.84 years)	ECG acquisition and HRV analysis/ultra-short HRV with LFn and HFn indices in k-users—changes in cardiac autonomic function with parasympathetic dominance, no link between HRV indices and k-use	No	No
[64]	To determine self-reported prevalence and severity of opioids AE after kratom initiation	(1) Observational design (cross-sectional study) (2) Convenience sampling (3) Illicit opioid users with current k-use (*N* = 163, M, 37.10 years) (4) ND	Face-to-face interview—semi-structured questionnaire—severity of opioids AE/reduced prevalence of opioids AE (respiratory depression, constipation, physical pain, insomnia, depression, loss of appetite, craving, decreased sexual performance, weight loss, fatigue) linked to kratom initiation	No	Yes, reported potential as an opioid substitute
[66]	To investigate the self-reported association between kratom initiation, regular use of illicit drugs, and HIV risk behaviors	(1) Observational (cross-sectional study) (2) Convenience sampling (3) Illicit drug users with current k-use (*N* = 260, M, 46 years) (4) ND	Face-to-face interview—semi-structured questionnaire/reduced regular use of illicit drugs (e.g., heroin, methamphetamine, amphetamine, cannabis, benzodiazepine, ketamine, methadone, and alcohol) and reduced HIV risk behaviors related to kratom initiation	No	Yes, reported potential as a form of harm reduction
[58]	To measure kratom dependence, withdrawal, and craving	(1) Observational design (cross-sectional survey) (2) Purposive sampling (3) Regular k-users (*N* = 293, M, 28.9 years) (4) ND	LDQ/severe (> 50%)-moderate dependence (45%) with physical and psychological withdrawal symptoms; MWC-MCQ short form/mainly (77%) low craving (with 23% high craving)	Yes, more severe dependence linked to prolonged k-use	No
[62]	To evaluate kratom effects on social functioning in the traditional setting	(1) Observational design (cross-sectional survey) (2) Snowball sampling (3) Regular k-users (*N* = 293, M, 28 years) (4) ND	ASI/no major impairments (despite kratom dependence) in social functioning in the traditional setting	No	Yes, authors believe this data might cautiously suggest kratom’s potential to substitute heroin in opiate addicts
[53]	To clinically investigate the testosterone levels following long-term k-use	(1) Observational design (cross-sectional study) (2) Snowball sampling (3) Regular k-users (*N* = 19, M, 30 years) (4) ND	Full-blood exams: testosterone, FSH, LH levels, biochemical content of iron, glucose, urea, creatinine, eGFR, calcium, inorganic phosphate, uric acid, sodium, potassium, chloride, TC, HDL, LDL, TG/HDL ratio, total protein, albumin, globulin, A/G ratio, total bilirubin, alkaline phosphatase, AST, ALT, ESR, RBC, Hb, PCV, MCV, MCH, platelet, WBC; % of neutrophil, lymphocyte, monocyte, eosinophil, and basophil cells/no alterations; creatinine, potassium and free T4 = slight differences within normal range; a slight increase in FSH-testosterone level with more than 3 glasses of k-use was reported, but values were within the normal range	No	Yes, authors believe that kratom might be a good medicine as it does not cause male hormonal alterations
[55]	To evaluate kratom’s effects on hematological and clinical-chemistry parameters	(1) Observational design (cross-sectional study) (2) Snowball sampling (3) Regular k-users (*N* = 58, M, 25.63 years) (4) Healthy subjects (*N* = 19, M, 28.91 years)	Face-to-face interview, full-blood exams/reduction in RBC, hemoglobin, and WBC in k-users within the normal range; slight changes (kidney, lipid, liver parameters) within normal range, higher HDL and slight LDL elevation (linked to prolonged and higher daily k-use)	No	Yes, increased HDL
[60]	To evaluate the severity of pain and sleep problems related to kratom withdrawal	(1) Observational design (cross-sectional study) (2) Snowball sampling (3) Regular k-users (*N* = 170, M, 31.7 years) (4) ND	BPI/moderate-severe (16%) pain intensity and moderate-severe interference (70–30%) during kratom cessation, more evident pain interference with higher doses (≥ 4 glasses); PSQI/moderate sleep problems (54%) during kratom cessation, associated with the quantity of k-use (higher with ≥ 4 glasses)	Yes	No
[59]	To assess the severity of anxiety and depression during kratom withdrawal	(1) Observational design (retrospective study) (2) Snowball sampling (3) Regular k-users (*N* = 150, M, 34.4 years) (4) ND	Face-to-face interview; BDI/mild depression (81% of participants) during kratom cessation linked to higher quantities (≥ 4 glasses of kratom daily); BAI/mild anxiety (70% of participants) during kratom cessation; no severe anxiety/depression linked to k-use	Yes	No
[54]	To investigate effects of long-term k-use on cognitive functioning	(1) Observational design (cross-sectional study) (2) Convenience sampling (3) Regular k-users (*N* = 70, M, 28.8 years) (4) Healthy subjects (*N* = 25, M, 25 years)	CANTAB—PAL/visual episodic memory-new learning impairment (no differences in terms of k-use); CANTAB—MOT, DMS, SWM, RTI, AST/no alterations	Yes/No	No
[57]	To investigate constipation prevalence k-use related and fatigue severity during k-use cessation	(1) Observational design (retrospective study) (2) Convenience sampling (3) Regular k-users (*N* = 125, M, 34 years) (4) ND	CAS/limited constipation problems (only 7 subjects) during k-use; FSS/severe fatigue (108 subjects) during kratom cessation linked to higher (3 or more glasses daily) k-use	Yes	No
[75]	To evaluate if k-use produces dose-dependent effects	(1) Observational design (cross-sectional study) (2) Convenience sampling (3) Regular k-users (*N* = 62, F = 1, 43.8) (4) ND	B-BAES/no significant differences in the stimulant and sedative domains based on the length of k-use or the assumed dose	Yes	No
[65]	To examine the motives for k-use and experiences in reducing HIV risk behaviors	(1) Observational design (cross-sectional study) (2) Convenience sampling (3) Out-of-treatment HIV-positive opiate users (*N* = 32, M, 41.7 years) with current k-use (*N* = 20) (4) ND	Semi-structured questionnaire/k-use for opioid withdrawal, increase energy, to substitute heroin, to reduce risk behaviors (e.g., sexual practices, injecting illicit substances, sharing the injecting equipment)	No	Yes, k-use reportedly helped to reduce opiate use, risky injecting and sexual behaviors
[71]	To assess the PK of the kratom	(1) Interventional design (prospective study) (2) Convenience sampling (3) Healthy previous k-users abstaining from kratom for several weeks (*N* = 7, F = 4, age range 26–40 years) (4) ND	2 g of kratom tea; vital signs (BP, oxygen saturation, pulse) + single blood draws at 24, 48, 72, 96, and 120 h after kratom tea administration; urine sample (0–12 h)/no severe AE, 2 subjects had lightheadedness/first blood draw and mild headache not related to kratom and continued the study; PK: oral two-compartment model	No	No
[70••]	To assess the PK of MG and its linearity	(1) Interventional design (Prospective experimental study) (2) ND (3) Chronic-regular k-users (*N* = 10, M, 27.1 years) (4) ND	MG p.o. 1 dose (6.25 mg) for 7 days + final dose (range 6.25—23 mg) at 8th day; observation/tongue numbness after having drunk kratom tea; blood exams and urine samples/no alteration; safety and vital signs/temporary increase of BP and pulse rate, no other signs; PK: oral two-compartment model	No	Yes, authors believe that kratom has a promising pharmacokinetic profile as pain killer or opioid substitute
[51]••	To estimate kratom’s effects on pain tolerance	(1) Interventional design (randomized placebo-controlled, double-blind study) (2) Sample size calculated for the proposed within-subject pilot study (3) Regular k-users (*N* = 26, M, 24.3 years) (4) ND	Participants received a kratom decoction drink 3 times during the study day—KPP, PKP, PPK (20 subjects) vs Placebo (PPP, 6 subjects); CPT/increased pain tolerance 1 h after kratom intake; blood exams and vital signs/no alterations; COWS/no discomfort or withdrawal for about twenty hours after discontinuation; subjective report of discomfort or unusual symptoms/none	No	Yes, increase in pain tolerance

*%*, percentage; *AE*, adverse effects; *ALP*, alkaline phosphatase; *ALT*, alanine transaminase; *ASI*, Addiction Severity Index; *AST* (CANTAB), attention switching task; *AST*, aspartate transaminase; *BAI*, Beck Anxiety Inventory; *B-BAES*, Brief-Biphasic Alcohol Effects Scale; *BDI*, Beck Depression Inventory; *BP*, blood pressure; *BPI*, Brief Pain Inventory; *BPRS*, Brief Psychiatric Rating Scale; *CANTAB*, Cambridge Neuropsychological Test Automated Battery; *CAS*, Constipation Assessment Scale; *COWS*, Clinical Opioid Withdrawal Scale; *CPT*, cold pressor task; *DB*, direct bilirubin; *DMS*, delayed matching to sample; *DSMV*, Diagnostic and Statistical Manual of Mental Disorders V Edition; *ECG*, electrocardiogram; *eGFR*, estimated glomerular filtration rate; *ESR*, erythrocyte sedimentation rate; *FG*, fasting glucose; *FSH*, follicle-stimulating hormone; *FSS*, Fatigue Severity Scale; *HB*, hemoglobin; *HDL*, high-density lipoprotein; *HFn*, high frequency; *HRV*, heart rate variability; *KDS*, Kratom Dependence Scale; *k-use*, kratom use; *k-users*, kratom users; *LDL*, low-density lipoprotein; *LDQ*, Leeds Dependence Questionnaire; *LFn*, normalized low frequency; *LH*, luteinizing hormone; *MCH*, mean corpuscular hemoglobin; *MCQ*, Marijuana Craving Questionnaire; *MCV*, mean corpuscular volume; *MetS*, metabolic syndrome; *MG*, mitragynine; *MINI*, Mini International Neuropsychiatric Interview; *MOT*, Motor Screening Task; *MS*, *Mitragyna speciosa*; *MWC*, Marijuana Withdrawal Checklist; *N*, number of subjects; *p.o.*, oral administration; *PAL*, paired associates learning; *PASP*, artery systolic pressure; *PCR*, creatinine ratio; *PCV*, packed cell volume; *PK*, pharmacokinetic parameters; *PSQI*, Pittsburgh Sleep Quality Index; *QoL*, quality of life; *RBC*, red blood cells; *RTI*, reaction time; *SWM*, spatial working memory; *TB*, total bilirubin; *TC*, total cholesterol; *TG*, triglycerides; *WBC*, white blood cells; *WC*, waist circumference; *WHOQOL-BREF*, World Health Organization Quality of Life-BREF

### Clinical Issues/Health Hazards Linked to Kratom Use

An evaluation of kratom’s clinical implications should also consider the reported kratom-related negative clinical consequences. A large number of case reports and case series have been questioning kratom’s safety profile, especially when ingested outside controlled settings. Issues raised include the risks of kratom dependence or addiction with withdrawal symptoms, cases of neonates with abstinence syndrome born from mothers with or without kratom withdrawal, and neurological and psychiatric manifestations [76–78]. Recent evidence has reported episodes of mental and psychological distress, especially when kratom is used with nonmedical opioids and methamphetamine [79]. Other negative consequences to be investigated further include endocrinological damages, dermatological manifestations, electrolytic and kidney alterations, hepatic and gastrointestinal injuries, and respiratory and cardiological health hazards (e.g., [80–83]). Concerning kratom’s effects on cardiological functioning, data is still inconsistent. Trakulsrichai et al. [70••] found a temporary increase in pulse rate and blood pressure in kratom users, and Leong Bin Abdullah et al. [73] described a condition of sinus tachycardia without alterations of QTc intervals. However, in another study, kratom use has been associated with a dose-dependent prolonged QTc interval [49], highlighting the need for more investigation on the cardiological impact of kratom use. The serotonin syndrome, the autonomic nervous system dysfunction, an undifferentiated shock, conditions of multi-organ dysfunction [84–86], overdoses, and fatalities (e.g., [87, 88] must also be considered (for a summary of the main kratom-related toxicities and their clinical presentations, see Table 3).

**Table 3:** Kratom-related toxicities and side effects according to case reports/series presentations. Evidence related to kratom-associated clinical negative consequences in the last 5 years (2018–2022) has been reported. *PRES*, posterior reversible leukoencephalopathy; *ARDS*, acute respiratory distress syndrome

Reference(s)	Main symptoms and clinical presentations
Kratom-related neurological issues	
[77, 89, 90]	Central nervous system depression and coma, (focal, bilateral, and generalized tonic–clonic) seizures, ataxia, PRES (headache, disorientation, aphasia, confusion), transient paralysis (lightheadedness, weakness, paralysis as catatonic state), intracerebral hemorrhage
Kratom-related psychiatric issues	
[91, 92]	Altered mental status, agitation, psychotic symptoms (visual and auditory hallucinations, delusions of grandeur, paranoid thoughts), confusion, irritability, reduction in motivation, withdrawal symptoms with obsessive thoughts, and suicidal or homicidal ideation
Kratom-related dependence/withdrawal syndrome	
[93, 94]	Craving, physical (runny nose, fatigue, dyspepsia, nausea, anorexia, diarrhea, diaphoresis, myalgia, rhinorrhea, lacrimation, arthralgia) and psychological symptoms (e.g., aggression, hostility/irritability, nervousness, restlessness, inability to work, sadness, emotional imbalance, insomnia, depression, anxiety, thoughts of self-harm, sedation, anhedonia, poor concentration, social isolation)
Kratom-related neonatal abstinence syndrome, with or without maternal abstinence syndrome	
[78, 95]	Muscle hypertonicity, sneezing, jittery, crying, increased muscle tone, tachypnea, hyperthermia, excessive sucking, irritability, sleeplessness, facial excoriations, feeding intolerance
Kratom-related acute intoxication, overdoses, and fatalities	
[87, 88, 96, 97]	Overdose symptoms (respiratory depression, neurological and psychiatric acute manifestations), dry mouth, dizziness, palpitations, tremors, and death (cardiorespiratory arrest, hypoxic brain damage, intramuscular hemorrhage of the tongue, pulmonary edema and congestion, brain edema, left ventricular hypertrophy, rhabdomyolysis, renal failure, transient non-ischemic reversible cardiomyopathy, pressure necrosis, hepatic injury, electrolytic disturbances, multi-organ dysfunction)
Kratom-related ARDS	
[98]	Cough and dyspnea
Kratom-related cardiological issues	
[81, 99]	Cardiac arrest, systolic dysfunction, ventricular fibrillation, palpitations, tachycardia, hypertension, prolonged QT interval
Kratom-related dermatological issues	
[80]	Photo-distributed hyper-pigmented patches, sparing the knuckles on both hands
Kratom-related electrolytic and renal issues	
[100–102]	Acute renal insufficiency with high serum creatinine and proteinuria, dangerous hyponatremia, hyperkalemia
Kratom-related endocrinological issues	
[82, 103]	Primary hypothyroidism, secondary hypogonadism (low energy, poor libido, elevation of serum prolactin)
Kratom-related hepatic issues	
[25, 83]	Cholestatic, hepatocellular, and/or mixed liver injury (jaundice, itching, abdominal pain, fever, pale stools, dark urine, fatigue pruritus, nausea, vomiting, diarrhea, weight loss, decreased appetite, scleral icterus, moderate ascites, muscle spasms, uncontrollable limb jerking, yawning)
Kratom-related gastrointestinal issues	
[104, 105]	Constipation, pan-colitis, nausea, vomiting, physical withdrawal symptoms
Other kratom-related toxicities	
[84]	Diaphoresis, flushing, aphasia, confusion, dysarthria, right facial droop, fever, hyperreflexia, clonus, tremors/serotonin syndrome
[106]	Circulatory shock, metabolic acidosis, hypoxia, symptoms of autonomic nervous system dysfunction
[86, 104]	Multi-organ dysfunction (e.g., rhabdomyolysis, lethargy, confusion, transient hearing loss, right lower extremity swelling, diffuse body aches, fatigue, generalized weakness, compartment syndrome, acute kidney injury, liver dysfunction, cardiomyopathy)
[107]	Intentional overdose and suicide attempt/suspected Fanconi syndrome from cadmium toxicity exacerbated by heavy kratom use
[85]	Undifferentiated shock and extreme elevation of procalcitonin/nausea, vomiting, weakness, and dizziness

While in Asia kratom has been used with a limited number of associated health hazards [25, 108], most of the safety issues have been reported in the West, mainly among users ingesting kratom on a daily basis in combination with other drugs, such as prescription medicines (e.g., antipsychotic, antidepressant, and hypnotic drugs, among others) and psychoactive substances (e.g., amphetamine, methamphetamine, cyclopropyl fentanyl, opioids, ethanol) [76, 88, 108]. Unscheduled contaminants/adulterants in kratom products (e.g., propyl-hexedrine, phenyl-ethylamine, O-desmethyl-tramadol, hydrocodone, and morphine) [90, 96•, 97•, 100], microbes (e.g., Salmonella, M. incognita), and toxic metals (lead and nickel) [109••, 110••, 111••] have also been found responsible for toxicities. Furthermore, MG was identified as a contributing factor to some fatalities [112]. More recently, a survey has suggested that kratom-related side effects might depend on the doses [113]. Thus, it is possible to hypothesize that low doses of kratom might come with smaller risks in the absence of other substances responsible for drug–drug interaction.

### Clinical Consequences of Kratom Use in Naïve Users

Limited information is available regarding kratom quantity used and clinical consequences in naïve users. Very few clinical reports of toxicity have been recorded among individuals using kratom alone and/or for the first time [77, 105]. With no clinical studies conducted among such populations, doubts remain about its safety. More rigorous research on the clinical, pharmacodynamic, and pharmacokinetic aspects of kratom and MG is needed to clarify the therapeutically useful or risky doses of kratom in humans.

## Discussion and Conclusion

With kratom research rapidly evolving, the most updated evidence on the clinical implications of kratom use has been reviewed in this work. Despite the growing amount of anecdotal self-reported evidence suggesting the therapeutic value of kratom in treating acute/chronic pain and psychiatric disorders, including SUD, in nonmedical settings, various studies report episodes of acute intoxications or the development of dependence linked to the heavy chronic use in such settings. Interestingly, most of the safety concerns derive from Western (non-native) countries, where kratom use frequently occurs in combination with other substances. Furthermore, kratom products are advertised online or elsewhere with captivating marketing strategies providing misleading or no information about the ingredients, dosage, type, and alkaloids, among others. These aspects make it difficult to determine the dose–response relationship and/or the causal relation with kratom exposure. On the contrary, most of the investigations that we found in native countries, mainly of cross-sectional nature, provide encouraging data on the potential therapeutic value of kratom, also supported by the initial preclinical evidence. Despite these discrepancies, it remains a priority to determine the dose of MG and other alkaloids that can be considered safe and clinically useful in medical treatment. Such insight might contribute to kratom’s risk assessment and give additional information to clinicians and regulatory agencies willing to recognize kratom as an agent for the treatment of pain, mental health, and other chronic/benign health conditions. Finally, controlled and longitudinal studies under careful clinical supervision, including healthy and naïve individuals and/or participants who consume other drugs or medicines outside the traditional context, will further contribute to the development of kratom research and provide a better understanding of the underlying clinical, pharmacological, and toxicological mechanisms necessary to inform future therapeutic applications of kratom.

## Data Availability

All the information that was used is presented in the tables and in the manuscript.
